# Design of an Angled Single-Excitation Elliptical Vibration System

**DOI:** 10.3390/mi16070808

**Published:** 2025-07-13

**Authors:** Qiang Liu, Xiping He, Weiguo Wang, Yanning Yang

**Affiliations:** 1 School of Physics and Electronic Information, Yan’an University, Yan’an 716000, China; wwgissp@126.com (W.W.); yadxyyn@163.com (Y.Y.); 2 School of Physics and Information Technology, Shaanxi Normal University, Xi’an 710119, China

**Keywords:** giant magnetostrictive material, single excitation, elliptical vibration, finite element calculation

## Abstract

An angled single-excitation elliptical vibration system for ultrasonic-assisted machining was developed in this paper, which was composed of a giant magnetostrictive transducer and an angled horn. Based on the continuous boundary conditions between the components, the frequency equation of the angled vibration system was derived, and the resonant frequencies of vibration systems with different angles were theoretically calculated. The finite element method was employed to investigate the impact of varying angles on the resonant frequency, elliptical trajectory, phase difference, and output amplitude of the vibration systems. The electrical impedance of the vibration system and the longitudinal and transverse vibration amplitudes at the end face of the horn were tested experimentally. The results show that the resonant frequency and phase difference in the vibration system decreased, the transverse amplitude of the output elliptical trajectory increased, and the longitudinal amplitude decreased with the increase in the included angle. The elliptical trajectories obtained from the test were generally consistent with the calculated results, and the calculated values of the resonant frequencies of the three angled vibration systems were in good agreement with the experimental test values.

## 1. Introduction

Ultrasonic elliptical vibration cutting is a machining process that fundamentally alters the cutting process by introducing elliptical vibration to the tool. Compared with ordinary cutting and onefoneng-dimensional ultrasonic vibration cutting, ultrasonic elliptical vibration cutting has the characteristics of intermittent cutting and friction reversal, which can reduce cutting force [1]; reduce tool wear; improve machining accuracy [2]; suppress burrs [3] and regenerative chatter [4]; prolong tool life [5]; and have many advantages such as high cutting stability, reduced surface roughness [6], and enhanced heat dissipation. It has good application prospects in precision and ultra-precision machining, as well as the processing of difficult-to-machine materials.

The ultrasonic elliptical vibration system is the key to achieving ultrasonic elliptical vibration cutting machining. Kurniawan et al. [7] proposed a three-dimensional ultrasonic elliptical vibration system driven by three sandwich piezoelectric transducers with amplitudes of 0.42 μm, 0.50 μm, and 0.60 μm in the three directions at a resonant frequency of 24 kHz, respectively. The feasibility of the vibration system was verified through micro-groove machining. The vibration trajectory adjustment range of the three-dimensional elliptical vibration system is small, and the trajectory adjustment is difficult. Hu et al. [8] developed a dual-excitation ultrasonic elliptical vibration system in which the longitudinal and transverse transducers were fixed at the node surface. The two transducers generated longitudinal vibrations in two directions, and the elliptical vibration trajectory was synthesized by combining these vibration components. Yang et al. [9] proposed an ultrasonic elliptical vibration cutting system with double longitudinal vibration excitation, in which two longitudinally vibrating piezoelectric transducers were positioned at an angle of 90°, generating an elliptical vibration trajectory with an adjustable shape in a working space of 5.1 μm × 5.3 μm. The vibration decoupling of the vibration system with a configuration angle of 90° (the included angle of two longitudinal transducers) is better than that with an acute angle. This layout state cannot introduce additional interference vibration modes in decoupling. The vibration system with a configuration angle of 90° can generate a standard elliptical trajectory. The elliptical vibration system consists of two transducers at a certain angle and fixed by a supporting structure, resulting in a larger spatial size. Bai et al. [10] developed an ultrasonic elliptical vibration system with a longitudinal bending composite mode, utilizing longitudinal vibration piezoelectric ceramics and bending vibration piezoelectric ceramics for driving. This adjustment of structural parameters aimed to align the natural frequencies of longitudinal and bending vibrations as closely as possible. The phase difference is a key parameter that affects the elliptical vibration trajectory of the output of the vibration system [11]. Under the excitation of two signals of the same frequency but different phases, the longitudinal vibration and bending vibration were superimposed to produce an elliptical vibration trajectory at the tip of the tool. Dual-excitation and three-dimensional elliptical vibration systems require multiple sets of driving elements to excite various vibration modes with the same resonant frequency, but the resonant frequencies of multiple groups of driving elements were difficult to adjust to the same frequency, and each group of driving elements must be equipped with a separate ultrasonic drive power signal, and the phase difference between the two ultrasonic drive power signals needs to be controlled. In this way, the structure of the ultrasonic vibration system and the control system becomes complex, and the control difficulty is high, resulting in unstable working performance, which restricts the application and promotion of ultrasonic elliptical vibration in practical industrial production.

Tan et al. [12] designed a single-excitation ultrasonic elliptical vibration cutting system with a tilted-slot structure, which was composed of a sandwich transducer with longitudinal vibration and a horn with a tilted-slot structure, and the horn converted the longitudinal vibration generated by the transducer into a longitudinal bending composite vibration and then generated elliptical vibration in the tool. The actual resonant frequency of the vibration system was 28.3 kHz, and the amplitudes in the axial and vertical directions were 8.7 μm and 6.8 μm, respectively, at an excitation voltage of 500 V_p-p_. The machining difficulty and cost of the tilted-slot elliptical vibration system were high, and the tilted slot had an impact on stiffness, which was not conducive to improving machining accuracy. Li et al. [13] attached a tool to one side of the transducer to create an asymmetrical structure and designed a single-excitation ultrasonic elliptical vibration transducer using the finite element method, with a resonant frequency of 22.5 kHz. Based on the theory of mechanical vibration, Yin et al. [14] developed a single-excitation ultrasonic elliptical vibration cutting device with a composite beam horn. The resonant frequency of the device was 20.1 kHz, and the cutting experiment of LY12 shows that elliptical vibration cutting can reduce the cutting force and the surface roughness of the workpiece. When the single-excitation elliptical vibration system was determined, the shape of the elliptical trajectory remained unchanged. The elliptical trajectory was a crucial factor influencing the surface quality of machining [15]. The amplitude and phase difference in the elliptical vibration trajectory have distinct effects on the roughness and surface micromorphology of the workpiece [16,17]. The development of an ultrasonic elliptical vibration system with adjustable vibration trajectory is of great significance for improving cutting performance and enhancing the quality of machined surfaces. Currently, transducers are categorized as piezoelectric transducers and magnetostrictive transducers according to the driving materials [18]. Compared to piezoelectric ceramics, giant magnetostrictive materials exhibit characteristics such as large magnetostrictive strain, high energy density, and lower sound velocity, with no permanent failure caused by overheating [19]. The giant magnetostrictive transducer has a broader bandwidth and a higher load force [20].

In this paper, based on the continuous boundary conditions between the components, the frequency equation of the angled elliptical vibration system was derived, and the resonant frequencies of the vibration systems with different angles were theoretically calculated. Finite element software was used to perform dynamic simulation calculations on the vibration system, and the effects of angles on the resonance frequency, phase difference, output amplitude, and elliptical trajectory of the vibration systems were studied. Three prototypes of the vibration systems were developed, and their vibration performance parameters were tested experimentally.

## 2. Structure of the Angled Elliptical Vibration System

**Figure 1:**
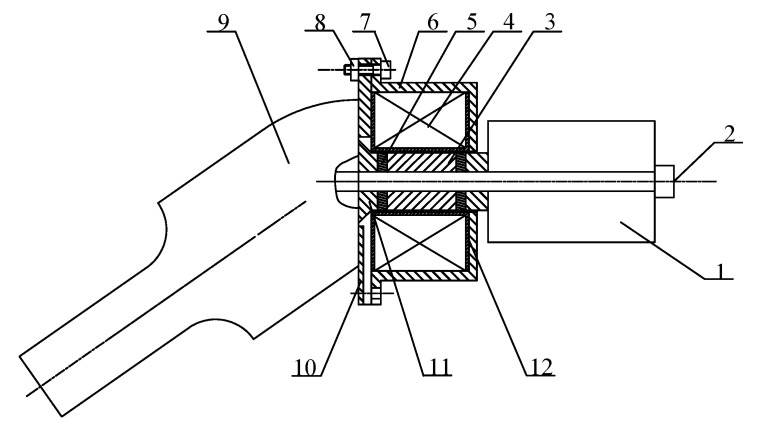
Design diagram of the angled elliptical vibration system: 1. tail mass, 2. preload bolts, 3. GMM rod, 4. coil, 5. permanent magnets, 6. magnetic cylinder, 7. bolt, 8. nut, 9. horn, 10. magnetic sheet, 11. magnetic blocks, and 12. coil skeleton.

Figure 1 shows the design diagram of the angled elliptical vibration system, which was composed of a giant magnetostrictive transducer and an angled horn. The giant magnetostrictive transducer mainly includes a magnetic block, a permanent magnet, a GMM rod, a coil, a tail mass, and a magnetic cylinder, with preload bolts connecting the transducer to the horn. The magnetic cylinder was arranged outside the coil to reduce the magnetic leakage of the transducer. The nodal surface was located at the joint surface of the horn and the magnetic block. The magnetic cylinder and the horn were fixed in place by bolts and nuts at the flange. After the coil of the transducer was energized, the GMM rod generated ultrasonic vibration in the alternating magnetic field generated by the coil, and transmitted the longitudinal vibration generated by the GMM rod to the horn. There is a certain angle between the horn and the transducer, which changes the transmission direction of the vibration generated by the transducer, and an elliptical composite vibration is then formed at the output end of the horn.

## 3. Theoretical Design

Ignoring the influence of rotational inertia, the longitudinal and transverse displacement of the particle in the rod can be in the following forms [21]:

(1) $$\varepsilon =A\cos k_{l}x+B\sin k_{l}x$$

(2) $$\eta =C\cosh k_{f}x+D\sin k_{f}x+E\cos k_{f}x+F\sin k_{f}x$$

where *A*, *B*, *C*, *D*, *E*, and *F* are undetermined coefficients.

The lengths of the rods 2 and 4 in the angled vibration system are *l*_2_ and *l*_4_, respectively, and the radii are *R*_2_ and *R*_4_. *θ* is the angle between the two rods. *φ*, *F_l_*, *F_f_*, and *M* are the angular, longitudinal forces, and moment experienced by the particles in the rod.

The longitudinal displacement, longitudinal force, transverse displacement, transverse force, rotation angle, and moment at both ends of the uniform rod are *ε*_2_, *F_l_*_2_, *η*_2_, *F_f_*_2_, *φ*_2_, *M*_2_, *ε*_4_, *F_l_*_4_, *η*_4_, *F_f_*_4_, *φ*_4_, and *M*_4_, respectively. Using the above formula, the vibration state of the straight rod can be expressed as follows:

$$\varepsilon_{2}=a_{11}\varepsilon_{4}+a_{12}F_{l4}+a_{13}\eta_{4}+a_{14}F_{f4}+a_{15}\phi_{4}+a_{16}M_{4}$$

$$F_{l2}=a_{21}\varepsilon_{4}+a_{22}F_{l4}+a_{23}\eta_{4}+a_{24}F_{f4}+a_{25}\phi_{4}+a_{26}M_{4}$$

$$\eta_{2}=a_{31}\varepsilon_{4}+a_{32}F_{l4}+a_{33}\eta_{4}+a_{34}F_{f4}+a_{35}\phi_{4}+a_{36}M_{4}$$

$$F_{f2}=a_{41}\varepsilon_{4}+a_{42}F_{l4}+a_{43}\eta_{4}+a_{44}F_{f4}+a_{45}\phi_{4}+a_{46}M_{4}$$

$$\phi_{2}=a_{51}\varepsilon_{4}+a_{52}F_{l4}+a_{53}\eta_{4}+a_{54}F_{f4}+a_{55}\phi_{4}+a_{56}M_{4}$$

$$M_{2}=a_{61}\varepsilon_{4}+a_{62}F_{l4}+a_{63}\eta_{4}+a_{64}F_{f4}+a_{65}\phi_{4}+a_{66}M_{4}$$

The boundary conditions for the rod are as follows:

$$\varepsilon_{2}=\varepsilon {|}_{x=l},\;F_{l2}=-SE\frac{\partial \varepsilon }{\partial x}{|}_{x=l},\;\eta_{2}=\eta {|}_{x=l},\;F_{f2}=EI\frac{\partial^{3}\eta }{\partial x^{3}}{|}_{x=l},\;\phi_{2}=-\frac{\partial \eta }{\partial x}{|}_{x=l},$$

$$M_{2}=-EI\frac{\partial^{2}\eta }{\partial x^{2}}{|}_{x=l},\;\varepsilon_{4}=\varepsilon {|}_{x=0},\;F_{l4}=-SE\frac{\partial \varepsilon }{\partial x}{|}_{x=0},\;\eta_{4}=\eta {|}_{x=0},\;$$

$$F_{f4}=EI\frac{\partial^{3}\eta }{\partial x^{3}}{|}_{x=0},\;\phi_{4}=-\frac{\partial \eta }{\partial x}{|}_{x=0},\;M_{4}=-EI\frac{\partial^{2}\eta }{\partial x^{2}}{|}_{x=0}.$$

Substituting Equations (1) and (2) into the boundary conditions to obtain each undetermined coefficient,

$$A=\varepsilon_{4},\;B=-\frac{F_{l4}}{ESk_{l}},\;C=\frac{\eta_{4}}{2}-\frac{M_{4}}{2EIk_{f}^{2}},\;D=-\frac{\phi_{4}}{2k_{f}}+\frac{F_{f4}}{2EIk_{f}^{3}},\;E=\frac{\eta_{4}}{2}+\frac{M_{4}}{2EIk_{f}^{2}},F=-\frac{\phi_{4}}{2k_{f}}-\frac{F_{f4}}{2EIk_{f}^{3}}.$$

Substituting the obtained undetermined coefficients into the boundary conditions to obtain the following:

$$\varepsilon_{2}=\varepsilon_{4}\cos k_{l}l-F_{l4}\frac{\sin k_{l}l}{ESk_{l}},\;F_{l2}=\varepsilon_{4}ESk_{l}\sin k_{l}l+F_{l4}\cos k_{l}l,$$

$$\eta_{2}=\eta_{4}\frac{\cosh k_{f}l+\cos k_{f}l}{2}+F_{f4}\frac{\sinh k_{f}l-\sin k_{f}l}{2EIk_{f}^{3}}-\phi_{4}\frac{\sinh k_{f}l+\sin k_{f}l}{2k_{f}}+M_{4}\frac{-\cosh k_{f}l+\cos k_{f}l}{2EIk_{f}^{2}},$$

$$F_{f2}=\eta_{4}\frac{EIk_{f}^{3}(\sinh k_{f}l+\sin k_{f}l)}{2}+F_{f4}\frac{\cosh k_{f}l+\cos k_{f}l}{2}-\phi_{4}\frac{EIk_{f}^{2}(\cosh k_{f}l-\cos k_{f}l)}{2}+M_{4}\frac{k_{f}(-\sinh k_{f}l+\sin k_{f}l)}{2},$$

$$\phi_{2}=\eta_{4}\frac{k_{f}(-\sinh k_{f}l+\sin k_{f}l)}{2}+F_{f4}\frac{k_{f}(-\cosh k_{f}l+\cos k_{f}l)}{2EIk_{f}^{3}}+\phi_{4}\frac{\cosh k_{f}l+\cos k_{f}l}{2}+M_{4}\frac{k_{f}(\sinh k_{f}l+\sin k_{f}l)}{2EIk_{f}^{2}},$$

$$M_{2}=\eta_{4}\frac{EIk_{f}^{2}(-\cosh k_{f}l+\cos k_{f}l)}{2}+F_{f4}\frac{-\sinh k_{f}l-\sin k_{f}l}{2k_{f}}-\phi_{4}\frac{EIk_{f}(-\sinh k_{f}l+\sin k_{f}l)}{2}+M_{4}\frac{\cosh k_{f}l+\cos k_{f}l}{2}.$$

The characteristic matrix of uniform rod vibration can be expressed as follows:

(3) $$\left[\begin{array}{l} \varepsilon_{2}\\ F_{l2}\\ \eta_{2}\\ F_{f2}\\ \phi_{2}\\ M_{2} \end{array}\right]=\left[\begin{array}{cccccc} a_{11} & a_{12} & 0 & 0 & 0 & 0\\ a_{21} & a_{22} & 0 & 0 & 0 & 0\\ 0 & 0 & a_{33} & a_{34} & a_{35} & a_{36}\\ 0 & 0 & a_{43} & a_{44} & a_{45} & a_{46}\\ 0 & 0 & a_{53} & a_{54} & a_{55} & a_{56}\\ 0 & 0 & a_{63} & a_{64} & a_{65} & a_{66} \end{array}\right]\left[\begin{array}{l} \varepsilon_{4}\\ F_{l4}\\ \eta_{4}\\ F_{f4}\\ \phi_{4}\\ M_{4} \end{array}\right]$$

where $a_{11}=\cos k_{l}l$, $a_{12}=-\frac{\sin k_{l}l}{ESk_{l}}$, $a_{21}=ESk_{l}\sin k_{l}l$, $a_{22}=\cos k_{l}l$, $a_{33}=\frac{\cosh k_{f}l+\cos k_{f}l}{2}$, $a_{34}=\frac{\sinh k_{f}l-\sin k_{f}l}{2EIk_{f}^{3}}$,

$$a_{35}=-\frac{\sinh k_{f}l+\sin k_{f}l}{2k_{f}},\;a_{36}=\frac{-\cosh k_{f}l+\cos k_{f}l}{2EIk_{f}^{2}},\;a_{43}=\frac{EIk_{f}^{3}(\sinh k_{f}l+\sin k_{f}l)}{2},$$

$$a_{44}=\frac{\cosh k_{f}l+\cos k_{f}l}{2},\;a_{45}=-\frac{EIk_{f}^{2}(\cosh k_{f}l-\cos k_{f}l)}{2},\;a_{46}=\frac{k_{f}(-\sinh k_{f}l+\sin k_{f}l)}{2},$$

$$a_{53}=\frac{k_{f}(-\sinh k_{f}l+\sin k_{f}l)}{2},\;a_{54}=\frac{k_{f}(-\cosh k_{f}l+\cos k_{f}l)}{2EIk_{f}^{3}},\;a_{55}=\frac{\cosh k_{f}l+\cos k_{f}l}{2},$$

$$a_{56}=\frac{k_{f}(\sinh k_{f}l+\sin k_{f}l)}{2EIk_{f}^{2}},\;a_{63}=\frac{EIk_{f}^{2}(-\cosh k_{f}l+\cos k_{f}l)}{2},\;a_{64}=\frac{-\sinh k_{f}l-\sin k_{f}l}{2k_{f}},$$

$$a_{65}=-\frac{EIk_{f}(-\sinh k_{f}l+\sin k_{f}l)}{2},\;a_{66}=\frac{\cosh k_{f}l+\cos k_{f}l}{2}.$$

At the connection between rod 2 and rod 4, the continuity of displacement, force, rotation angle, and moment can be expressed in matrix form as follows:

(4) $$\left[\begin{array}{l} \varepsilon_{2}\\ F_{l2}\\ \eta_{2}\\ F_{f2}\\ \phi_{2}\\ M_{2} \end{array}\right]=\left[\begin{array}{cccccc} \cos\theta & 0 & \sin\theta & 0 & 0 & 0\\ 0 & \cos\theta & 0 & \sin\theta & 0 & 0\\ -\sin\theta & 0 & \cos\theta & 0 & 0 & 0\\ 0 & -\sin\theta & 0 & \cos\theta & 0 & 0\\ 0 & 0 & 0 & 0 & 1 & 0\\ 0 & 0 & 0 & 0 & 0 & 1 \end{array}\right]\left[\begin{array}{l} \varepsilon_{4}\\ F_{l4}\\ \eta_{4}\\ F_{f4}\\ \phi_{4}\\ M_{4} \end{array}\right]$$

**Figure 2:**
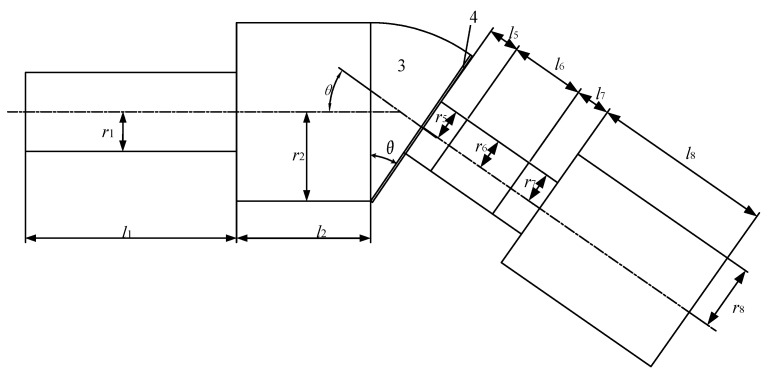
Simplified schematic diagram of the vibration systems.

Figure 2 shows a simplified schematic diagram of the angled elliptical vibration system. According to the different materials and cross-sectional sizes of each part of the vibration system, it was divided into eight parts.

Let the longitudinal displacement, longitudinal force, transverse displacement, transverse force, rotation angle, and moment on the left side of part *i* be *ε_i_*, *F_li_*, *η_i_*, *F_fi_*, *φ_i_*, and *M_i_*, respectively. The longitudinal displacement, longitudinal force, transverse displacement, transverse force, rotation angle, and moment on the right side of part *i* be *ε_i_*_+1_, *F_li_*_+1_, *η_i_*_+1_, *F_fi_*_+1_, *φ_i_*_+1_, and *M_i_*_+1_, respectively.

The vibration characteristic equation in part *i* can be expressed as follows:

(5) $$\left[\begin{array}{l} \varepsilon_{i}\\ F_{li}\\ \eta_{i}\\ F_{fi}\\ \phi_{i}\\ M_{i} \end{array}\right]=\left[\begin{array}{cccccc} a_{11}^{i} & a_{12}^{i} & a_{13}^{i} & a_{14}^{i} & a_{15}^{i} & a_{16}^{i}\\ a_{21}^{i} & a_{22}^{i} & a_{23}^{i} & a_{24}^{i} & a_{25}^{i} & a_{26}^{i}\\ a_{31}^{i} & a_{32}^{i} & a_{33}^{i} & a_{34}^{i} & a_{35}^{i} & a_{36}^{i}\\ a_{41}^{i} & a_{42}^{i} & a_{43}^{i} & a_{44}^{i} & a_{45}^{i} & a_{46}^{i}\\ a_{51}^{i} & a_{52}^{i} & a_{53}^{i} & a_{54}^{i} & a_{55}^{i} & a_{56}^{i}\\ a_{61}^{i} & a_{61}^{i} & a_{63}^{i} & a_{64}^{i} & a_{65}^{i} & a_{66}^{i} \end{array}\right]\left[\begin{array}{l} \varepsilon_{i+1}\\ F_{li+1}\\ \eta_{i+1}\\ F_{fi+1}\\ \phi_{i+1}\\ M_{i+1} \end{array}\right]$$

(6) $$\begin{array}{l} \left[\begin{array}{cccccc} a_{11}^{i} & a_{12}^{i} & 0 & 0 & 0 & 0\\ a_{21}^{i} & a_{22}^{i} & a_{23}^{i} & a_{24}^{i} & a_{25}^{i} & a_{26}^{i}\\ a_{31}^{i} & a_{32}^{i} & a_{33}^{i} & a_{34}^{i} & a_{35}^{i} & a_{36}^{i}\\ a_{41}^{i} & a_{42}^{i} & a_{43}^{i} & a_{44}^{i} & a_{45}^{i} & a_{46}^{i}\\ a_{51}^{i} & a_{52}^{i} & a_{53}^{i} & a_{54}^{i} & a_{55}^{i} & a_{56}^{i}\\ a_{61}^{i} & a_{61}^{i} & a_{63}^{i} & a_{64}^{i} & a_{65}^{i} & a_{66}^{i} \end{array}\right]=\left[\begin{array}{cccccc} a_{11}^{1} & a_{12}^{1} & 0 & 0 & 0 & 0\\ a_{21}^{1} & a_{22}^{1} & 0 & 0 & 0 & 0\\ 0 & 0 & a_{33}^{1} & a_{34}^{1} & a_{35}^{1} & a_{36}^{1}\\ 0 & 0 & a_{43}^{1} & a_{44}^{1} & a_{45}^{1} & a_{46}^{1}\\ 0 & 0 & a_{53}^{1} & a_{54}^{1} & a_{55}^{1} & a_{56}^{1}\\ 0 & 0 & a_{63}^{1} & a_{64}^{1} & a_{65}^{1} & a_{66}^{1} \end{array}\right]\\ \cdot \cdot \cdot \cdot \cdot \cdot \left[\begin{array}{cccccc} a_{11}^{8} & a_{12}^{8} & 0 & 0 & 0 & 0\\ a_{21}^{8} & a_{22}^{8} & 0 & 0 & 0 & 0\\ 0 & 0 & a_{33}^{8} & a_{34}^{8} & a_{35}^{8} & a_{36}^{8}\\ 0 & 0 & a_{43}^{8} & a_{44}^{8} & a_{45}^{8} & a_{46}^{8}\\ 0 & 0 & a_{53}^{8} & a_{54}^{8} & a_{55}^{8} & a_{56}^{8}\\ 0 & 0 & a_{63}^{8} & a_{64}^{8} & a_{65}^{8} & a_{66}^{8} \end{array}\right] \end{array}$$

where the coefficient of the matrix [*a^i^*] is determined by the material properties of the *i*th part, as well as the length and cross-sectional area, and the displacement, force, angle of rotation, and moment between the parts of the vibration system are equal. Therefore, the relationship between the output end face of the horn and the rear end face of the tail mass can be expressed as follows:

(7) $$\left[\begin{array}{l} \varepsilon_{0}\\ F_{l0}\\ \eta_{0}\\ F_{f0}\\ \phi_{0}\\ M_{0} \end{array}\right]=\left[\begin{array}{cccccc} A_{11} & A_{12} & A_{13} & A_{14} & A_{15} & A_{16}\\ A_{21} & A_{22} & A_{23} & A_{24} & A_{25} & A_{26}\\ A_{31} & A_{32} & A_{33} & A_{34} & A_{35} & A_{36}\\ A_{41} & A_{42} & A_{43} & A_{44} & A_{45} & A_{46}\\ A_{51} & A_{52} & A_{53} & A_{54} & A_{55} & A_{56}\\ A_{61} & A_{61} & A_{63} & A_{64} & A_{65} & A_{66} \end{array}\right]\left[\begin{array}{l} \varepsilon_{8}\\ F_{l8}\\ \eta_{8}\\ F_{f8}\\ \phi_{8}\\ M_{8} \end{array}\right]$$

The total matrix of the vibration system [*A*] is the product of the matrices of each part, that is,

(8) $$\left[A\right]=\left[a^{1}\right]\left[a^{2}\right]\left[a^{3}\right]\left[a^{4}\right]\left[a^{5}\right]\left[a^{6}\right]\left[a^{7}\right]\left[a^{8}\right]$$

The rear surface of the tail mass and the output end of the horn were free boundary conditions, and the longitudinal force, transverse force, and moment at both ends were zero, that is,

$$F_{l0}=F_{l8}=F_{f0}=F_{f8}=M_{0}=M_{8}=0$$

Substituting the above relationship into Equation (8), the frequency equation of the angled elliptical vibration system can be obtained as follows:

(9) $$\left|\begin{array}{ccc} A_{21} & A_{23} & A_{25}\\ A_{41} & A_{43} & A_{45}\\ A_{61} & A_{63} & A_{65} \end{array}\right|=0$$

The total length of the magnetic block and the permanent magnet at both ends of the GMM rod is *l*_5_ = *l*_7_ = 8 mm; the diameter of the GMM rod, the magnetic block, and the permanent magnet are *D*_5_ = *D*_6_ = *D*_7_ = 18 mm; the length of the GMM rod is *l*_6_ = 21 mm; the diameter of the tail mass is *D*_8_ = 38 mm; the length is *l*_8_ = 52 mm; and the size of each part of the angled horn is *D*_1_ = 23 mm, *l*_1_ = 63 mm, *D*_2_ = 52 mm, *l*_2_ = 38 mm, *D*_4_ = 52 mm, *l*_4_ = 2 mm. Table 1 shows the material parameters of the vibration systems. According to the frequency equation, Equation (9), and the material parameters of each part, the resonant frequencies of the vibration systems at different angles were calculated using Mathematica, as shown in Table 2.

## 4. Finite Element Calculation

**Figure 3:**
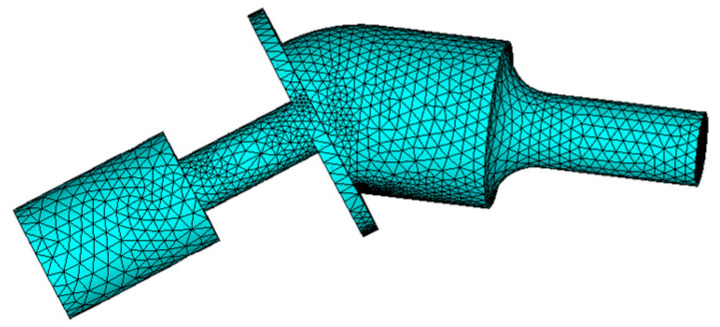
Grid diagram of the 35° vibration system.

**Figure 4:**
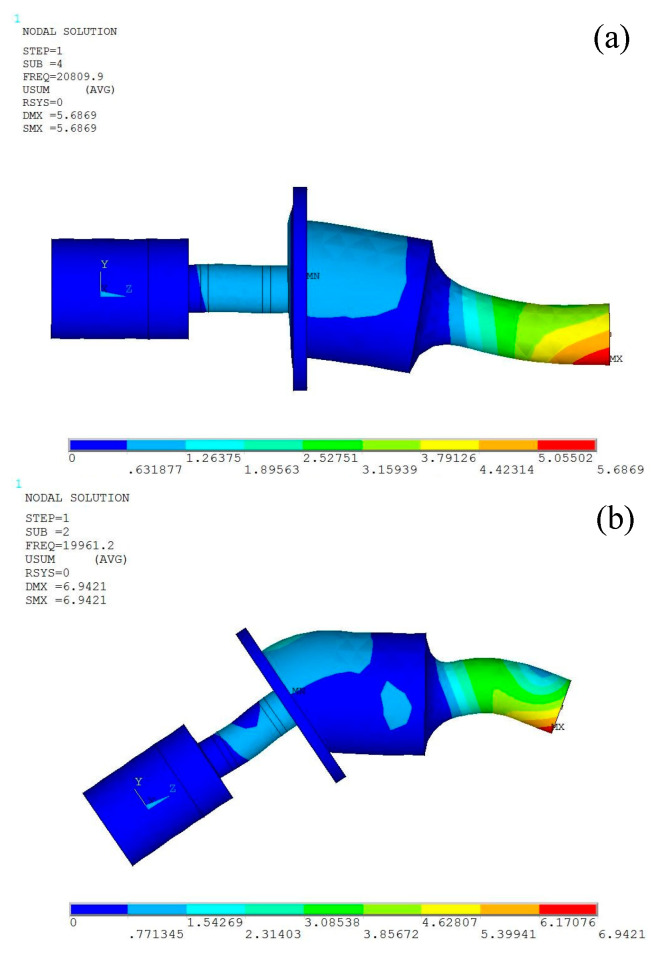

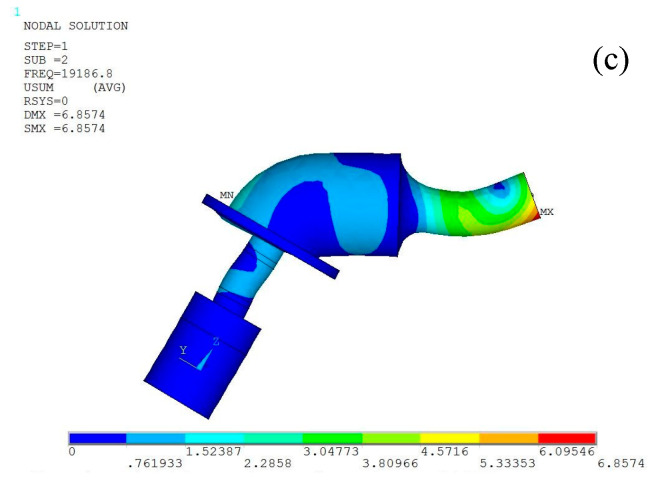
Vibration modes of vibration systems: (**a**) 10°, (**b**) 35°, and (**c**) 60°.

The model of the angled elliptical vibration system was established in SolidWorks 2012 and imported into the ANSYS 15.0 finite element software for modal analysis. The Solid98 element was used for the GMM rod, and the Solid95 element was used for the other parts of the vibration systems. Free meshing was selected to mesh the vibration system model; the number of nodes and elements of the model were 86,609 and 56,528, respectively, and the frequency range was set from 15 to 25 kHz. Figure 3 shows the grid diagram of the 35° vibration system. The vibration system can output elliptical trajectories in the range of 10~60°, and the three angles selected within this range were 10°, 35°, and 60°. Figure 4 shows the vibration modes of the angled elliptical vibration system. The resonant frequencies of the vibration systems at angles of 10°, 35°, and 60° were 20.810 kHz, 19.961 kHz, and 19.187 kHz, respectively. The output end of the horn exhibits both longitudinal vibration and bending vibration. The amplitude of the output end of the horn was the largest, while the amplitudes of the flange and the tail mass were relatively small.

**Figure 5:**
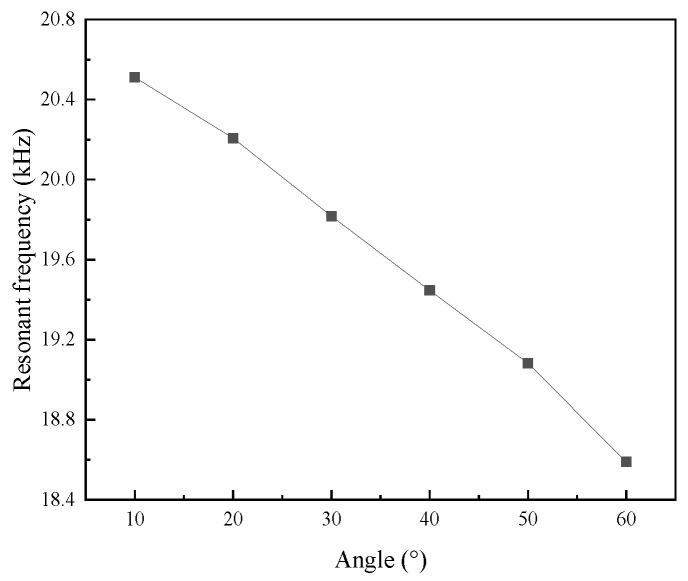
The resonant frequency of vibration systems with different angles.

Figure 5 shows the resonant frequency of the vibration systems at different angles. The increase in the angle of the vibration system is equivalent to an increase in effective length, resulting in a decrease in its resonant frequency.

**Figure 6:**
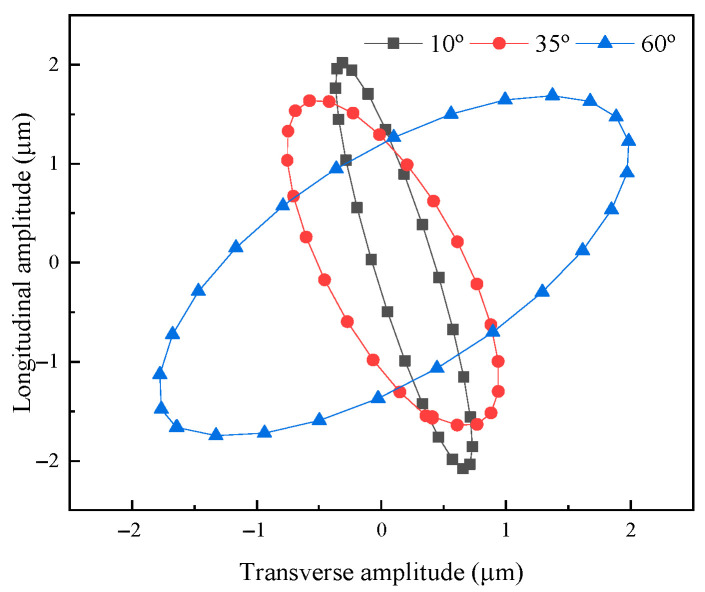
Elliptical trajectory of vibration systems with different angles.

Transient analysis was conducted on elliptical vibration systems with angles of 10°, 35°, and 60°. The material parameters are in Table 1. The sine excitation voltage was 250 V, and the transient analysis of 10 vibration cycles was carried out. The longitudinal and transverse output amplitudes were extracted at the output end of the horn, and the elliptical trajectory was obtained by curve fitting, as shown in Figure 6.

**Figure 7:**
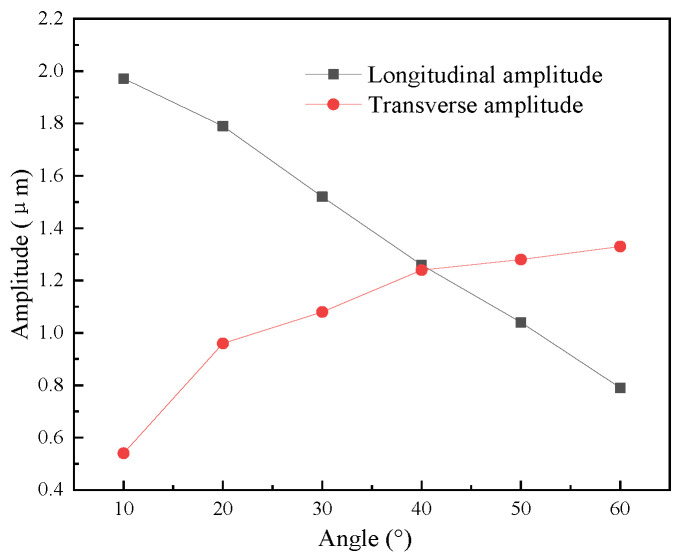
Output amplitude of the vibration systems with different angles.

**Figure 8:**
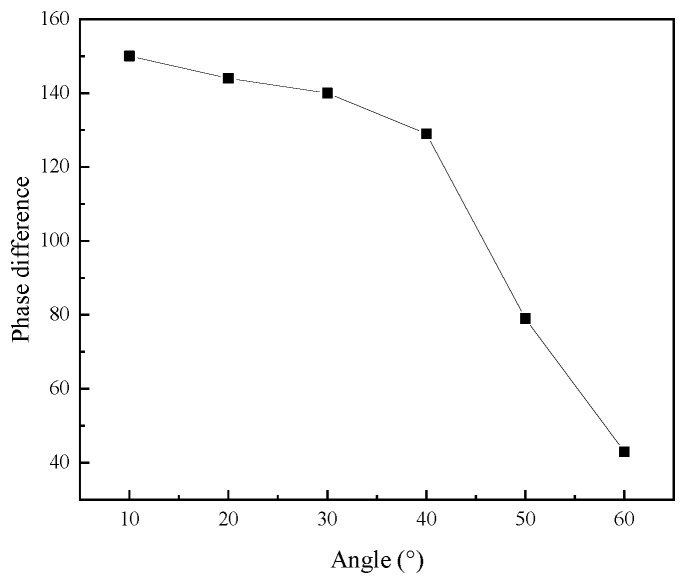
Phase difference in the vibration systems with different angles.

Figure 7 shows the output amplitude of the elliptical vibration system with different angles. It can be seen that as the angle increased, the transverse amplitude of the output elliptical trajectory of the vibration system increased, and the longitudinal amplitude decreased. Figure 8 shows the phase difference in the elliptical vibration system with different angles. As the angle increased, the phase difference decreased.

## 5. Experimental Test

**Figure 9:**
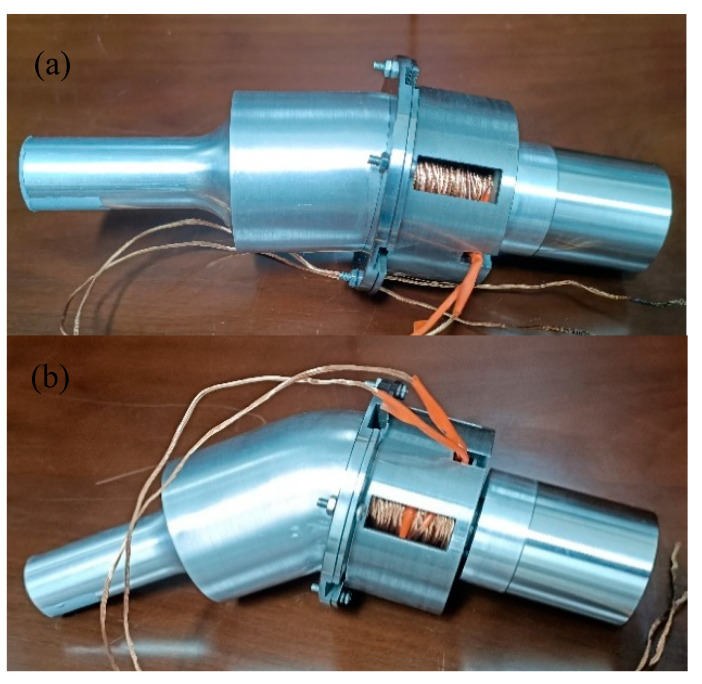

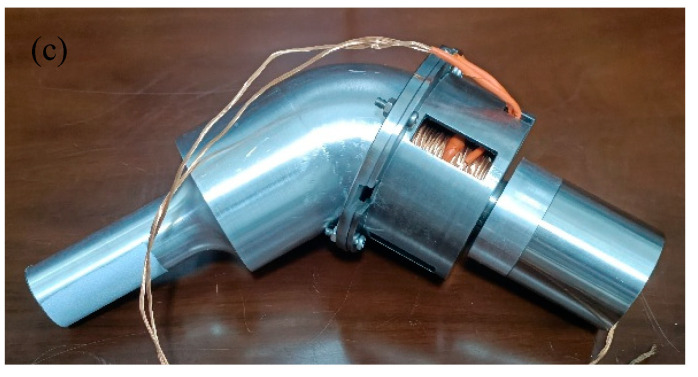
Prototype of three vibration systems at (**a**) 10°, (**b**) 35°, and (**c**) 60°.

**Figure 10:**
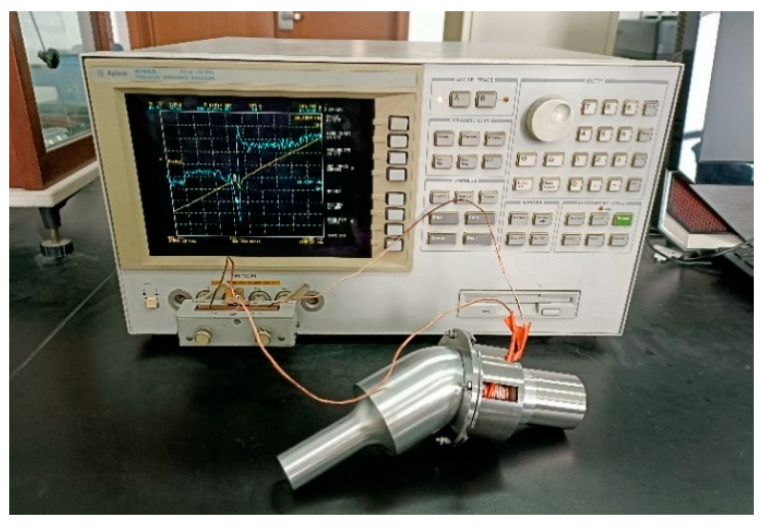
Impedance test diagram of the vibration systems.

Figure 9 shows the prototypes of three angled elliptical vibration systems. The angles of the vibration systems were 10°, 35°, and 60°, respectively. The impedance of the elliptical vibration system was experimentally tested using an impedance analyzer (4294A, Agilent, Santa Rosa, CA, USA), as shown in Figure 10.

**Figure 11:**
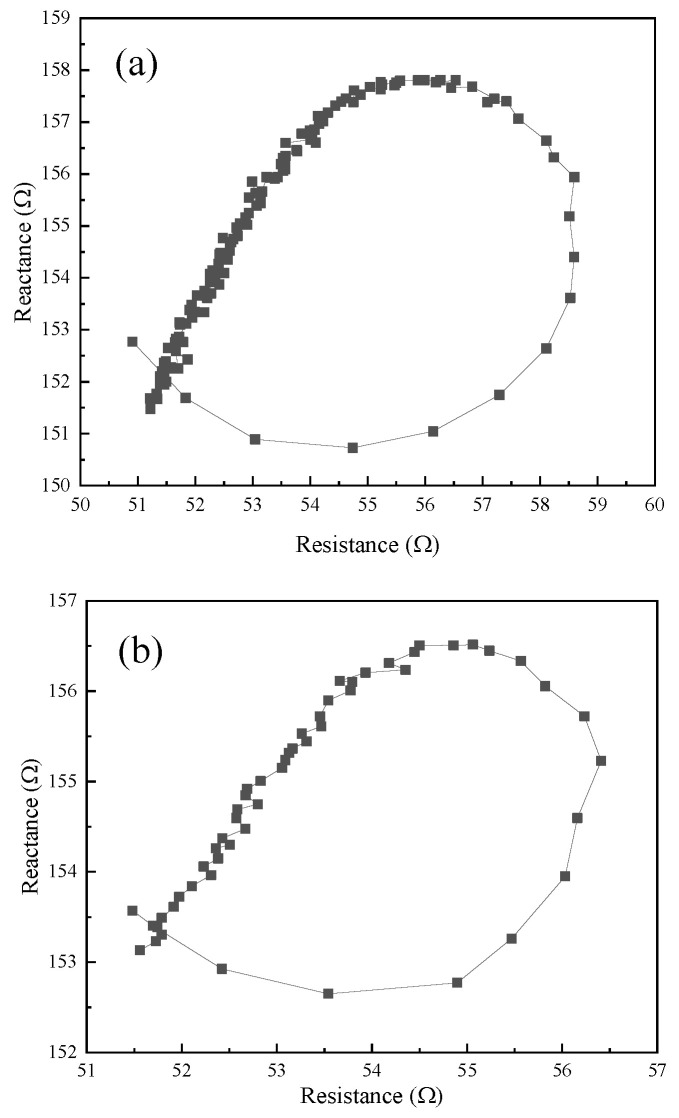

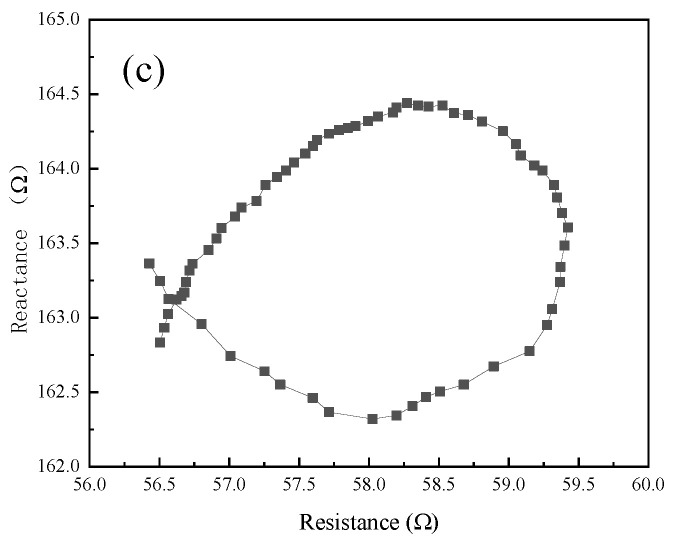
Impedance circle of the vibration systems at (**a**) 10°, (**b**) 35°, and (**c**) 60°.

Figure 11 shows the impedance circle of three angled elliptical vibration systems. The resonant frequencies of the vibration systems with angles of 10°, 35°, and 60° were 20.38 kHz, 19.86 kHz, and 18.61 kHz, respectively. The mechanical quality factors of the three angled vibration systems were 88.61, 132.40, and 66.46, respectively.

Table 2 shows the actual test values, finite element calculation values, and theoretical calculation values of the resonant frequencies for the elliptical vibration systems with the three angles. According to the theoretical calculation, the resonant frequency of the vibration system was *f_a_*, the finite element simulation calculation was *f_n_*, and the experimental test was *f_m_*. Δ*f_a_* (%) was the relative error of the resonant frequency of the vibration system obtained by the theoretical calculation and experimental test, and Δ*f_n_* (%) was the relative error of the resonant frequency of the vibration system obtained by the finite element simulation calculation and experimental test. As shown in Table 2, the resonant frequency of the vibration systems decreased as the angle increased. There are certain errors between the theoretically calculated value of the resonant frequency, the simulated value, and the experimental test value. The reason may be that an error exists between the material parameters of the theoretical calculation and those of the actual vibration system. The manufacturing, processing, and assembly processes of the vibration system yield deviations from the theoretically calculated and simulated values. In addition, to reduce the stress concentration of the horn, setting a transition arc in the transition between the large end and the small end of the horn will also have a certain effect on the resonant frequency of the vibration systems.

**Figure 12:**
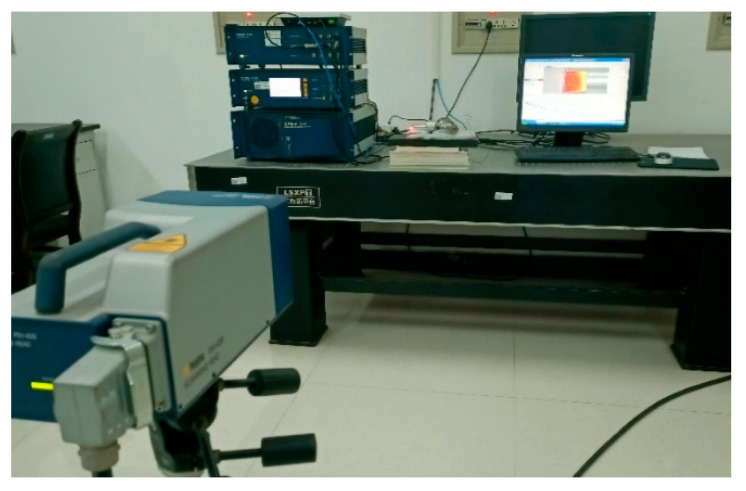
Experimental test diagram of the vibration systems.

A scanning laser vibrometer (PSV-400, Polytec, Karlsruhe, Germany) was used to test the mode shapes at the output ends of the horns of the three angled elliptical vibration systems, as shown in Figure 12.

**Figure 13:**
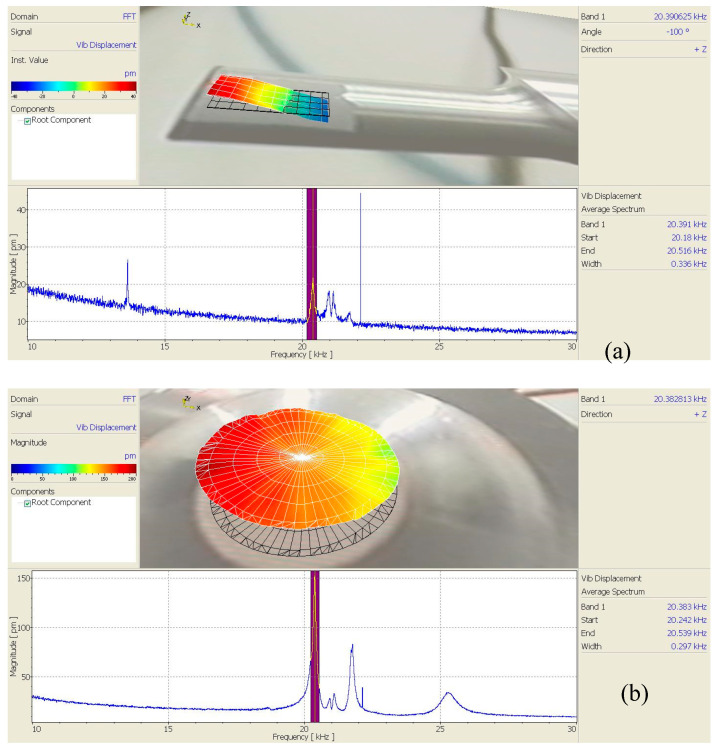
Vibration modes of the 10° angled vibration system: (**a**) side face and (**b**) end face.

**Figure 14:**
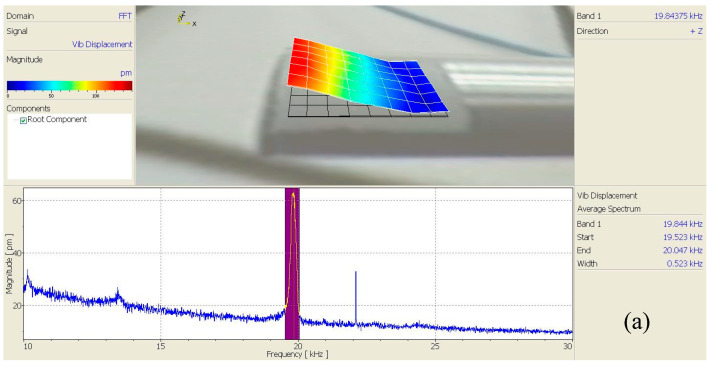

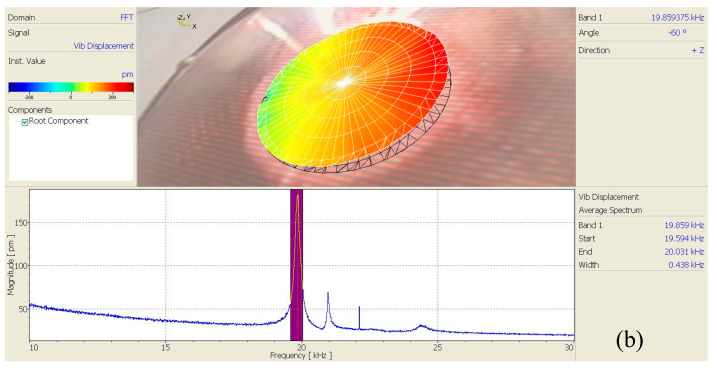
Vibration modes of the 35° angled vibration system: (**a**) side face and (**b**) end face.

**Figure 15:**
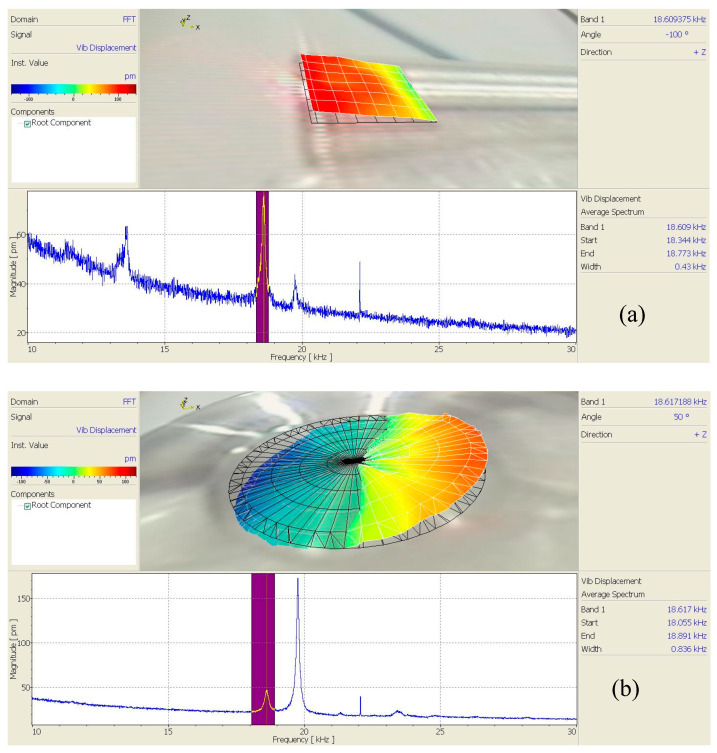
Vibration modes of the 60° angled vibration system: (**a**) side face and (**b**) end face.

Figure 13, Figure 14 and Figure 15 show the vibration model of the output end of the three angled vibration systems. It can be seen from the figure that the vibration modes of the end face (longitudinal) and side face (transverse) were bending vibrations. The longitudinal vibration resonance frequency and the bending vibration resonance frequency of the 10° angled vibration system were 20.383 kHz and 20.391 kHz, respectively, and the deviation value of the two was 8 Hz. The longitudinal vibration resonance frequency and the bending vibration resonance frequency of the 35° angled vibration system were 19.859 kHz and 19.844 kHz, respectively, and the deviation value of the two was 15 Hz. The longitudinal vibration resonance frequency and the bending vibration resonance frequency of the 60° angled vibration system were 18.617 kHz and 18.609 kHz, respectively, and the deviation value of the two was 8 Hz. The longitudinal vibration resonance frequency and bending vibration resonance frequency of the three angled vibration systems were relatively close, and the bending vibrations of the end face increased gradually as the angle increased.

**Figure 16:**
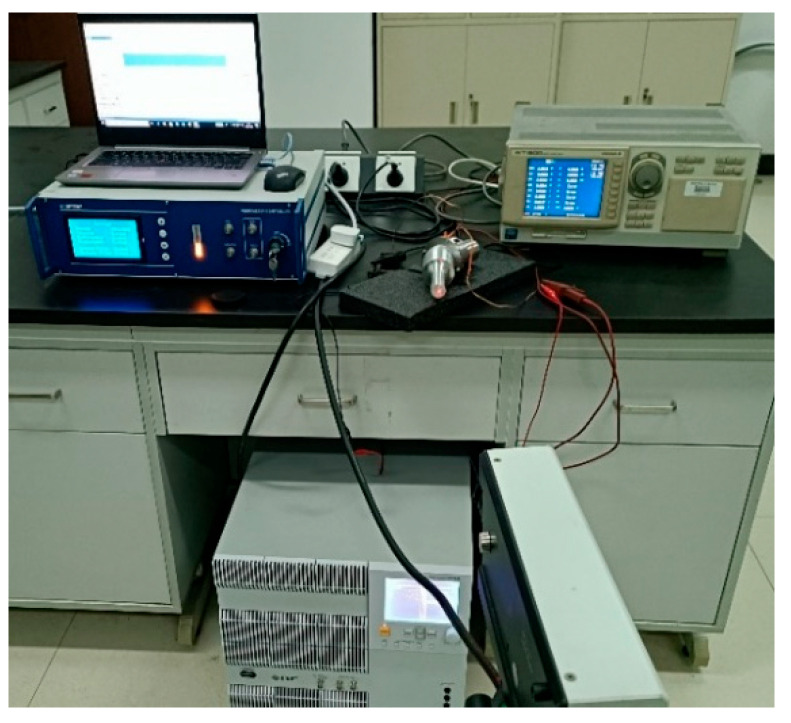
Amplitude test of the angled vibration systems.

The high-speed bipolar power supply (BP4620, NF, Kanagawa, Japan) was used to output high-frequency electrical signals, and the amplitudes of the end face and side face of the three angled elliptical vibration systems were measured using a laser vibrometer (LV-S01, Sunny Optical, Zhejiang, China). Figure 16 shows an experimental test diagram of the vibration systems.

**Figure 17:**
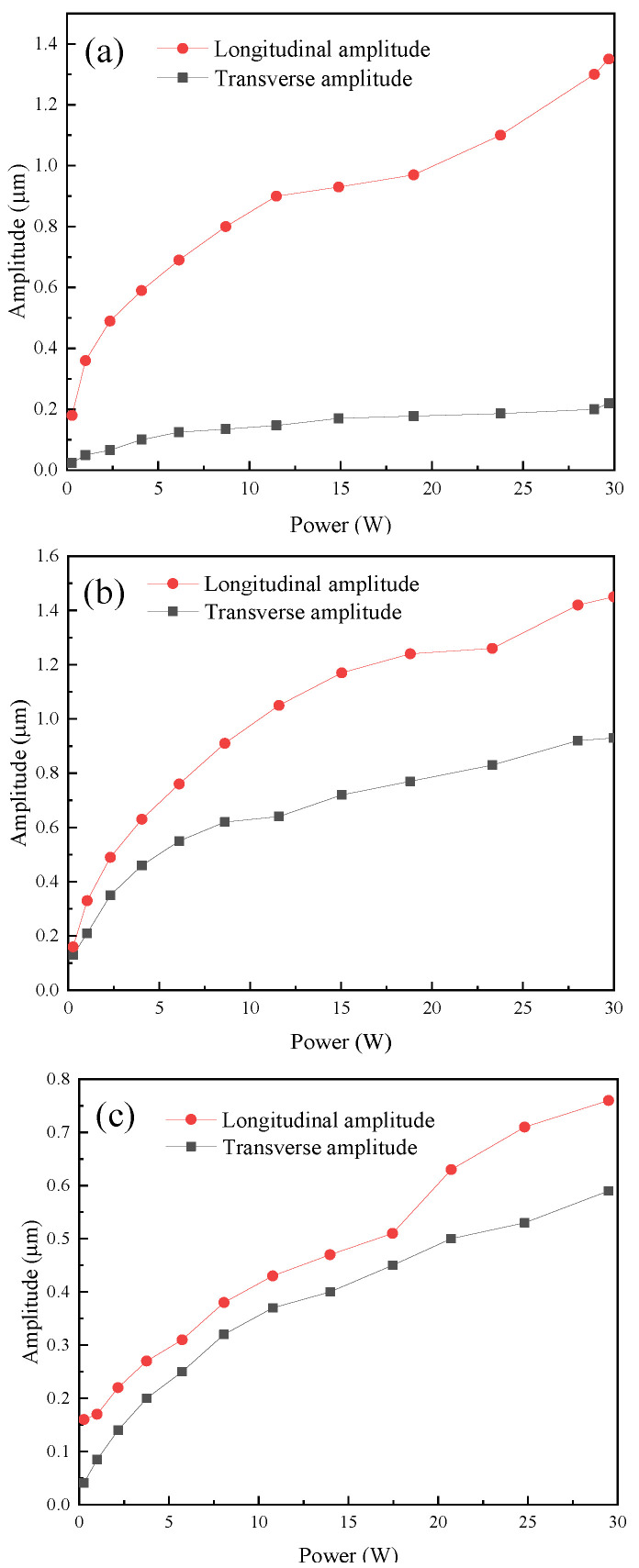
Power–amplitude curves of the angled elliptical vibration systems at (**a**) 10°, (**b**) 35°, and (**c**) 60°.

Figure 17 shows the effective power and output amplitude curves of the three angled elliptical vibration systems. It can be seen from the figure that with the increase in effective power, the longitudinal amplitude and transverse amplitude of the three angled vibration systems also increased. As the angle increased, the transverse amplitude of the vibration systems increased, while the longitudinal amplitude decreased.

**Figure 18:**
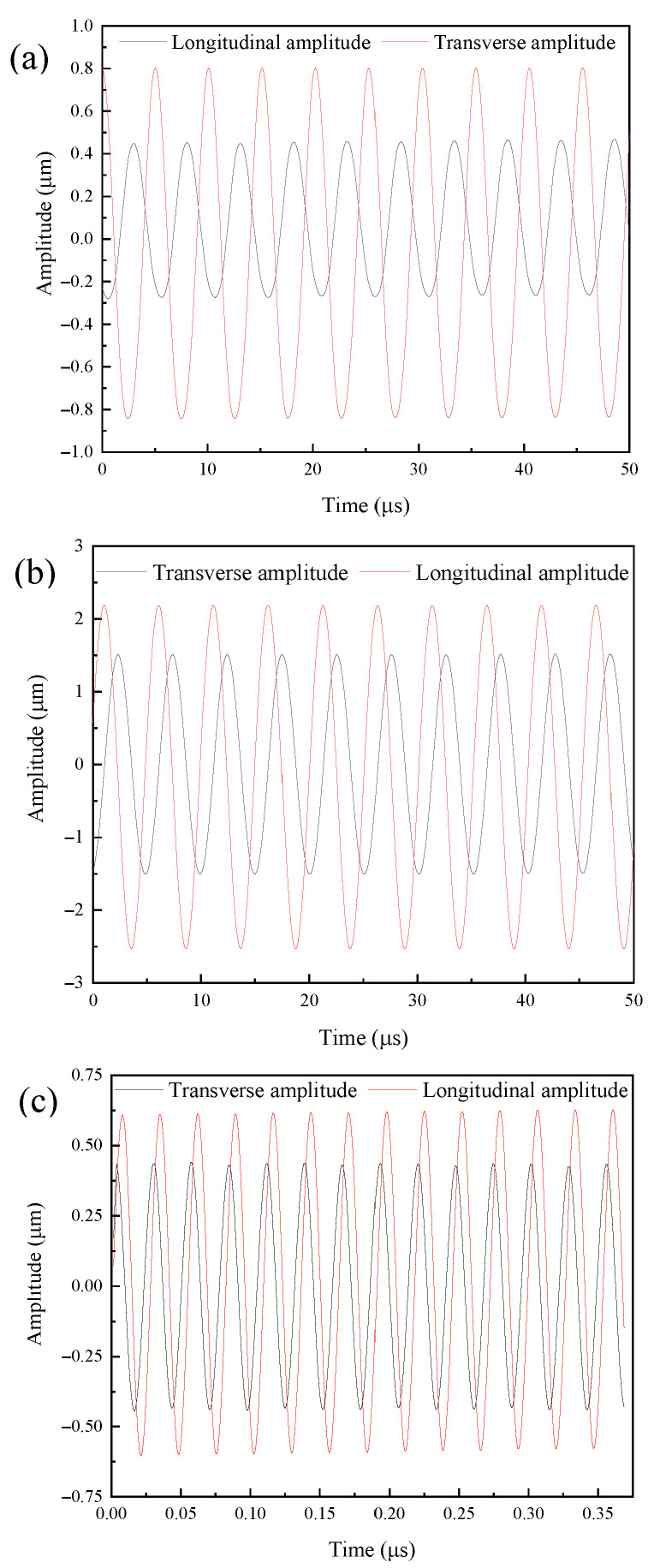
Time-domain amplitude curve of the vibration systems at (**a**) 10°, (**b**) 35°, and (**c**) 60°.

**Figure 19:**
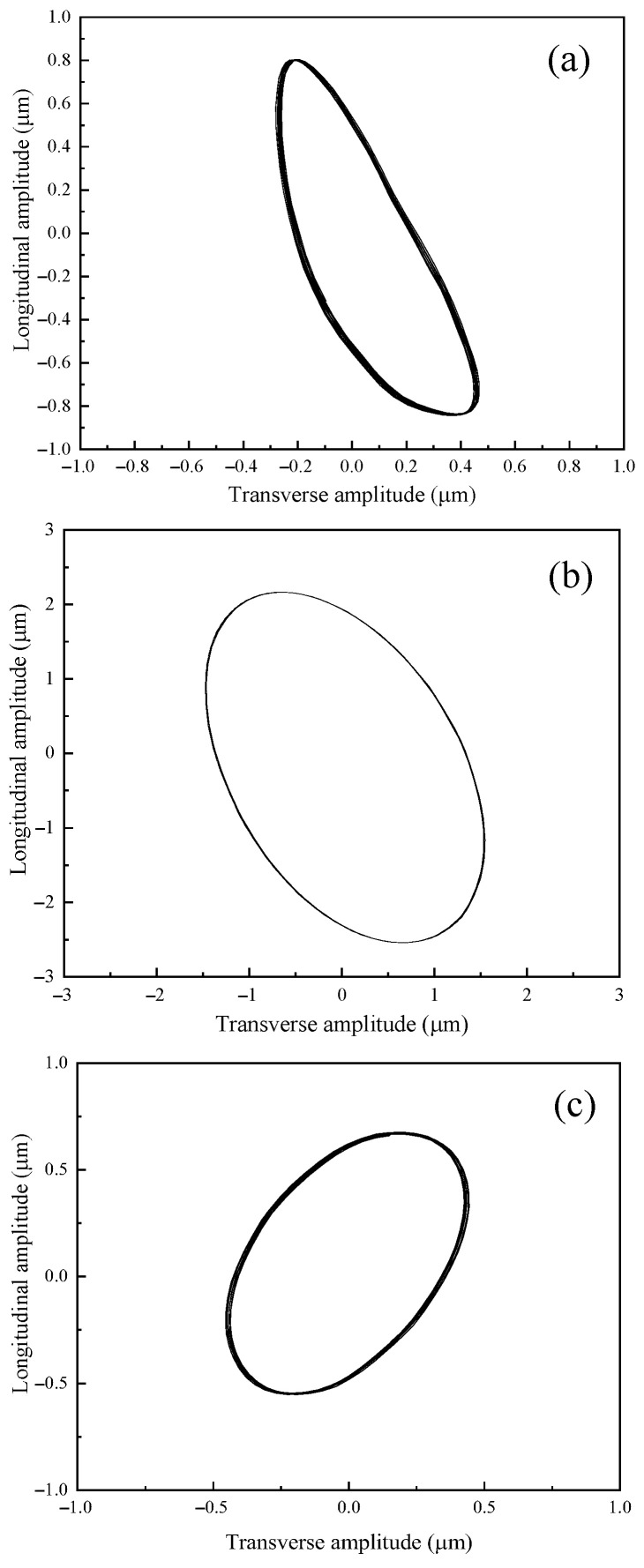
The elliptical trajectory of the angled elliptical vibration systems at (**a**) 10°, (**b**) 35°, and (**c**) 60°.

Figure 18 shows the amplitude curve of the output end of the vibration systems. It can be seen from the figure that the longitudinal vibration trajectory and transverse vibration trajectory of the output end were both stable sinusoidal curves. The elliptical trajectory was obtained by curve fitting the amplitude of the output end of the three angled elliptical vibration systems from the experimental test, as shown in Figure 19.

## 6. Conclusions

In this work, a single-excitation elliptical vibration system was developed, comprising a giant magnetostrictive transducer and an angled horn. The finite element method was employed to investigate the impact of angles on the resonant frequency, phase difference, elliptical trajectory, and output amplitude of the angled vibration systems. As the angle increased, the resonant frequency and phase difference in the vibration system decreased, the transverse amplitude of the output elliptical trajectory increased, and the longitudinal amplitude decreased.

The electrical impedance of three developed vibration systems and the longitudinal and transverse amplitudes at the output end of the horn were tested; the resonant frequencies of the vibration systems with angles of 10°, 35°, and 60° were 20.38 kHz, 19.86 kHz, and 18.61 kHz, respectively, and the mechanical quality factors were 88.61, 132.40 and 66.46, respectively. The elliptical trajectory obtained from the test was consistent with the calculated results. The theoretical calculation values, simulation calculation values, and experimental test values of the resonant frequency of the three-angled elliptical vibration system were generally consistent. The elliptical vibration systems developed in this paper can be utilized for different ultrasonic-assisted machining applications.

## Figures and Tables

**Table 1:** Material parameters.

Name	Material	Density (kg/m^3^)	Poisson’s Ratio	Young’s Modulus (GPa)
Magnetic Conductor	Pure iron	7860	0.29	195
GMM rod	Tb0.3Dy0.7Fe1.9	9250	0.30	27.50
Horn	Aluminum alloy	2790	0.34	71.50
Permanent magnet	NdFeB	7500	0.24	160
Tail mass	Stainless steel	8000	0.28	193

**Table 2:** The resonant frequency of the vibration systems.

Angle	*f_a_*/kHz	*f_n_*/kHz	*f_m_*/kHz	Δ*f_a_* (%)	Δ*f_n_* (%)
10°	21.10	20.81	20.38	3.53	2.11
35°	21.09	19.96	19.86	6.19	0.50
60°	21.05	19.19	18.61	13.11	2.96

## Data Availability

The data that support the findings of this study are available from the corresponding authors upon reasonable request.

## Nomenclature

Ignore the effects of shear deformation and rotational inertia effects:

*ε*	the longitudinal displacement
*η*	the transverse displacement
*θ*	the angle between the two rods
*R*	the radio
*S*	the cross-sectional area
*C*	the speed of sound
*E*	the Young’s modulus
*ω*	the angular frequency
$k_{l}=\omega /c$	the longitudinal wave number
kf=ρSω2EI14	the bending wave
I=r2S4	the moment of inertia of the circular section
$\phi =-\frac{\partial \eta }{\partial x}$	the rotation angle
$F_{l}=-ES\frac{\partial \varepsilon }{\partial x}$	the longitudinal force
$F_{f}=EI\frac{\partial^{3}\eta }{\partial x^{3}}$	the transverse force
$M=-EI\frac{\partial^{2}\eta }{\partial x^{2}}$	the moment
